# Improving Emergency Department Airway Preparedness in the Era of COVID-19: An Interprofessional, In Situ Simulation

**DOI:** 10.21980/J8V06M

**Published:** 2020-07-15

**Authors:** Keiran J Warner, Ashley C Rider, James Marvel, Michael A Gisondi, Kimberly Schertzer, Kelly N Roszczynialski

**Affiliations:** * Stanford University, Department of Emergency Medicine, Palo Alto, CA

## Abstract

**Audience:**

The target audience for this airway simulation includes all emergency department (ED) staff who are potential members of a COVID-19 intubation team, including emergency medicine attendings, emergency medicine residents, nurses, respiratory therapists, pharmacists, and ED technicians.

**Introduction:**

As of May 7, 2020 there were 1,219,066 diagnosed cases of COVID-19 in the U.S. and 73,297 deaths.^1^ A special report from the Centers for Disease Control and prevention on infections in healthcare personnel reported 9,282 cases between February 12^th^ and April 9th.^2^ Sars-CoV-2 is a novel virus that requires a careful, coordinated approach to airway management given the high risk of aerosolization.^3^ It is essential to train ED staff (1) to appropriately care for patients with suspected COVID-19 disease and (2) to provide an organized, safe working environment for providers during high-risk, aerosolizing procedures such as intubation. In addition to providing a set of airway management guidelines, we aimed to educate the staff through participation in a simulation activity. Due to the multiple team members involved and the array of equipment needed, an in-person in situ strategy was implemented. The goals of the simulation were to optimize patient care and minimize viral exposure to those involved during intubation.

**Educational Objectives:**

At the conclusion of the simulation session, learners will be able to: 1) Understand the need to notify team members of a planned COVID intubation including: physician, respiratory therapist, pharmacist, nurse(s), and ED technician. 2) Distinguish between in-room and out-of-room personnel during high-risk aerosolizing procedures. 3) Distinguish between in-room and out-of-room equipment during high-risk aerosolizing procedures to minimize contamination. 4) Appropriately select oxygenation therapies and avoid high-risk aerosolizing procedures. 5) Manage high risk scenarios such as hypotension or failed intubation and be prepared to give push-dose vasoactive medications or place a rescue device such as an I-gel ^®^.

**Educational Methods:**

This is a high-fidelity, interprofessional, in-situ simulation used to train a team of providers that would normally participate in the management of a patient with suspected COVID-19 requiring endotracheal intubation. Participants might include emergency medicine attendings, emergency medicine residents, nurses, respiratory therapists, pharmacists, and ED technicians. The patient is best represented by a high-fidelity mannequin such as Trauma HAL^®^ (Miami, FL USA) https://www.gaumard.com/products/trauma/trauma-halr), with a monitor displaying vital signs and voice-response capabilities. The simulation includes an interprofessional debriefing session, during which an airway checklist, communication strategies, and best practices are reviewed.

**Research Methods:**

Airway management guidelines were developed by an interdisciplinary team at our institution. We used these guidelines from Stanford Health Care and best practices from a literature review to create a checklist of recommended steps. Two assessors used the checklist to track team actions. Any missed items were discussed in the team debrief and participants were encouraged to ask questions. At the end of the session, to check for understanding, participants were provided with a brief anonymous online survey accessed by a QR code. These assessments allowed the simulation team to iteratively edit the case before future simulations.

**Results:**

From 3/23/20–4/23/20, we held 12 in-situ simulations with 62 participants, including emergency medicine physicians, nurses, technicians, respiratory therapists, and pharmacists. Two individuals observed each simulation and compared team performance to the checklist of recommended steps. The actions that were not completed during the simulation served as teaching points during the simulation debrief. The debrief discussions helped to identify misconceptions regarding oxygenation strategies, difficulties in staff communication due to physical barriers, and various other quality or safety concerns. Participant reactions following the simulation and debriefs were overwhelmingly positive.

**Discussion:**

This simulation was an effective, easy-to-implement method of interprofessional team training for a risk-inherent procedure in the ED. We learned that the deliberate simulation of each step of the COVID19-specific intubation procedure with all team members provided opportunities to identify safety challenges in communication, equipment, and approach. Each debrief stimulated an excellent discussion among team members, and allowed for interprofessional feedback, clarification of questions, and recommendations for areas of improvement. Our main take-away from the pilot of this novel simulation case is that new, high-risk procedures require a coordinated team effort to minimize risks to patients and staff, and that team training is feasible and effective using frequent in situ simulations.

**Topics:**

Medical simulation, in-situ simulation, interprofessional, COVID-19, novel coronavirus, SARS-CoV-2, intubation, medical education, health professions education, team training, airway management.

## USER GUIDE

[Table t1-jetem-5-3-s28]

**Table t1-jetem-5-3-s28:** 

List of Resources:	
Abstract	28
User Guide	30
Instructor Materials	32
Operator Materials	41
Debriefing and Evaluation Pearls	43
Simulation Assessment	45

**Learner Audience:**

Senior Residents, Attendings, Respiratory therapists, Pharmacists, Nurses, ED Techs

**Time Required for Implementation:**

Instructor Preparation: 30 minutes for set up of mannequin and props

Time for case: 20–30 minutes

Time for debriefing: 10 minutes

**Recommended Number of Learners per Instructor:**

One attending, one resident, two nurses, one pharmacist, one technician, and one respiratory therapist for a total of up to seven learners per case. If one of these participants cannot attend, a confederate may perform the actions of that healthcare professional.

**Topics:**

Medical simulation, in-situ simulation, interprofessional, COVID-19, novel coronavirus, SARS-CoV-2, intubation, medical education, health professions education, team training, airway management.

**Objectives:**

After completion of the simulation, learner will be able to:

1. Understand the need to notify the team members of a planned COVID intubation including the physician, respiratory therapist, pharmacist, nurse(s), and ED technician.
2. Distinguish between in-room and out-of-room personnel during high-risk aerosolizing procedures. In-room personnel includes 1–2 physicians, a respiratory therapist, and one nurse. All other staff remain outside of the room.
3. Distinguish between in-room and out-of-room equipment during high-risk aerosolizing procedures to minimize contamination.
4. Review oxygenation therapies for COVID patients and understand the need to avoid unnecessary aerosolizing procedures such as bagging.
5. Review management of high-risk scenarios such as hypotension or failed intubation and be prepared to give push-dose vasoactive medications or place a rescue device such as an I-gel^®^.

### Linked objectives and methods

This format allows for execution of each of these steps among an interprofessional team. The in-situ interprofessional simulation format was selected because it allows participants to appreciate the interdependence between team members. In addition, it was important to hold the simulation in the ED setting where these intubations occur, in order to enhance realism and allow for identification of systems issues. The interprofessional nature and setting helped to accomplish the first two learning objectives, which involve recruitment of the entire team and establishing in-room and out-of-room personnel. Participants had departmental guidelines available to them during the simulation, which facilitated familiarization prior to using the guidelines for a real patient. These guidelines helped the teams in collecting the appropriate equipment, which accomplished objective three. In the debrief, any misunderstanding about the steps of the procedure were discussed. Special attention was given to the oxygenation strategies during the scenario, and if interventions such as bagging or non-invasive ventilation were used by the team, they were discussed as inappropriate methods of oxygenation in order to highlight objective four. Finally, to assess objective five, the scenario involved a complication such as hypotension or hypoxemia in order for the team to practice integrating rescue medications or placing rescue airway devices. After the scenario, the interprofessional team debrief discussion allowed for identification of systemic quality issues related to the five objectives that otherwise may have been encountered during actual patient care.

### Recommended pre-reading for instructor

Note: these references were recommended at the time of submission, but this is subject to change with rapidly evolving information about the virus.

- Alhazzani W, Møller MH, Arabi YM, et al. Surviving sepsis Campaign: guidelines on the management of critically ill adults with coronavirus disease 2019 (COVID-19). *Intensive Care Med.* 2020;46(5):854–887. doi:10.1097/CCM.0000000000004363.
- Cook TM, El-Boghdadly K, McGuire B, McNarry AF, Patel A, Higgs A. Consensus guidelines for managing the airway in patients with COVID-19: guidelines from the Difficult Airway Society, the Association of Anaesthetists, the Intensive Care Society, the Faculty of Intensive Care Medicine and the Royal College of Anaesthetists. *Anaesthesia* 2020; 75(6):785–799. doi:10.1111/anae.15054.
- Giwa AL, Desai A, Duca A. Novel 2019 coronavirus SAR-SCoV- 2 (COVID-19): an overview for emergency clinicians. *Pediatr Emerg Med Pract*. 2020;17(5):1–24.
- Kovacs G, Sowers N, Campbell S, French J, Atkinson P. Just the facts: airway management during the COVID-19 pandemic. *CJEM.* 2020;1–5. doi:10.1017/cem.2020.353.

Websites:

- PPE donning and doffing: https://www.cdc.gov/hai/pdfs/ppe/ppe-sequence.pdf. Accessed 6/30/2020.
- NIH critical care guidelines: https://www.covid19treatmentguidelines.nih.gov/introduction/. Updated May 12^th^. Accessed 6/30/2020.
- Collection of resources from HIPPO Education: https://covid.hippoed.com/. Accessed 6/30/2020.

### Learner responsible content (optional)

Learners should be familiar with their own institutional guidelines for intubation of suspected COVID-19 patients. In addition, review of references listed above is optional based on learner role and interest.

### Associated content (optional)

- Check for understanding survey example
- COVID Airway Team Performance Checklist (note this checklist has 26 items instead of 28 due to the removal of two items that were institution-specific in an effort to make the checklist more generic)

### Results and tips for successful implementation

From 3/23/20–4/23/20, we held 12 in-situ simulations with 62 participants, including emergency medicine physicians, nurses, technicians, respiratory therapists, and pharmacists. Two individuals observed each simulation for completion of checklist items. The average performance on the checklist was 89% (range 75%-100%), or three items incorrect on a checklist of 28 items. The actions that were not completed during the simulation served as teaching points during the simulation debrief. Commonly missed items included obtaining a weight early on to facilitate the pharmacist’s preparation of medications and attaching the bag valve mask to oxygen prior to rapid sequence intubation.

The simulations provided an opportunity to clarify misconceptions regarding recommended oxygenation strategies in COVID-19 patients, thereby minimizing potential aerosolization. The debriefs identified difficulties in staff communication through the relatively soundproof isolation room doors, resulting in increased use of available video conferencing between in-room and outside-room providers. Various technical challenges with the procedure were addressed such as appropriate use of viral filters, communicating medication dosing with outside-room pharmacists, use of outside-room interpreters, exchanging airway equipment for smaller patients, safely obtaining radiographs through the exam room glass doors, and optimally placing personnel and equipment in the room. Additionally, we were able to identify several quality and safety concerns that were subsequently addressed with the appropriate individuals.

This is ideally implemented as an on-shift in situ simulation during lower volume hours in the ED. We found that 6am was the best time to run the scenario with the night shift team before signing out, and 8am was the best time to run the scenario with the day shift team. It was important that all team members were represented to ensure a successful simulation. If one could not make it due to a patient care obligation, the simulation instructors filled in as confederates. It was critical to provide a pre-brief to the team with introductions of each participant, orientation to the mannequin, and expectations for the scenario, including verbalizing (rather than donning) personal protective equipment. We found the simulation duration varied between 18–30 minutes, so it was important to respect participants’ time by keeping the debrief to less than 10 minutes. We created a case-specific checklist to track critical steps that teams performed or didn’t perform in order to address these steps during the debrief. A short objective-based survey was provided to inform authors of any modifications needed during future simulations. Finally, there was ample time for discussion about quality improvement or safety issues, and the simulation team escalated any recommendations to the appropriate leadership.

## Supplementary Information





## Figures and Tables

**Table t2-jetem-5-3-s28:** 

#1	iSTAT machine showing initial VBG (venous blood gas) (created on small cardboard box) (If desired, not essential)
#2	An electrocardiogram (EKG) printed demonstrating sinus tachycardia (printed) (If desired, not essential)

**Table t3-jetem-5-3-s28:** 

**Vitals:**	Heart Rate (HR) 122	Blood Pressure (BP) 108/66	Respiratory Rate (RR) 38
	Temperature (T) 102.2°F (36.7°C)	Oxygen Saturation (O_2_Sat) 76% (6L)	

**Figure f1-jetem-5-3-s28:**
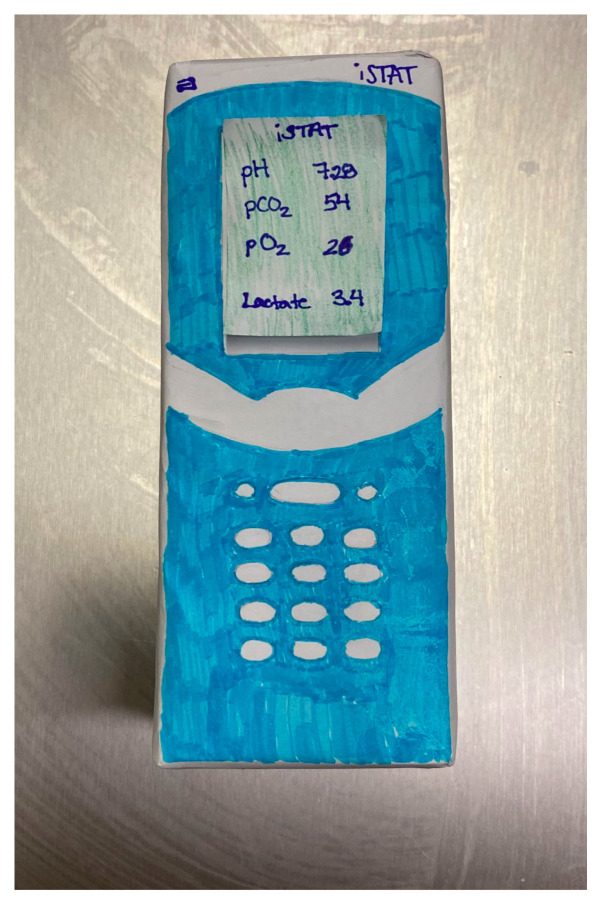


**Figure f2-jetem-5-3-s28:**
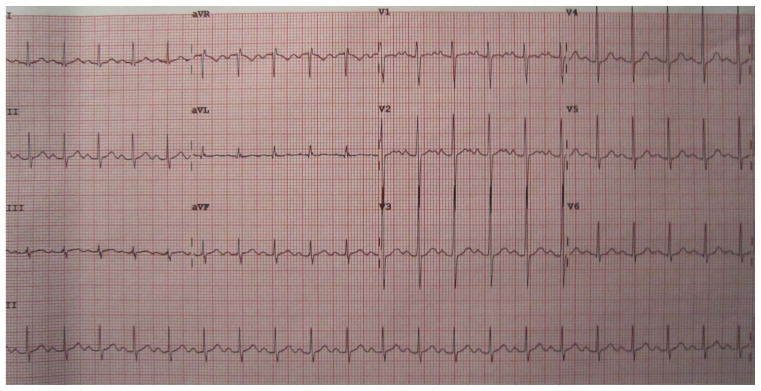


**Table t4-jetem-5-3-s28:** SIMULATION EVENTS TABLE:

Minute (state)	Participant action/trigger	Patient status (simulator response) & operator prompts	Monitor display (vital signs)
0:00 (Baseline)	Case begins. Patient is placed on cardiac monitor and EMS gives handoff to bedside RN.	Bedside RN recognizes the patient as a COVID PUI and does an initial assessment with appropriate PPE verbalized. RN recognizes respiratory distress and escalates care by alerting physicians and placing the patient on NRB for low O2 saturation. Patient is tachypneic and hypoxic on 6L NC to 81%.	T 102.1F HR 122 BP 108/65 RR 38 O2 79%–81% on 6L NC
05:00	Bedside RN gives report to physicians.	Physicians recognize that the patient is a COVID PUI and in severe respiratory distress. After escalating to NRB he continues to be in significant distress and more confused. At this time if the team attempts HFNC or NIPPV, the patient will further decompensate. Physician orders labs, COVID testing, ECG, i-STAT (if applicable), and gathers team for intubation including attending, respiratory therapy, pharmacy, second RN, and ED tech.	T 102.1F HR 118 BP 110/62 RR 40 O2 85% on 15L NRB
10:00	Team is gathered and airway checklist performed outside of room.	ED tech gathers airway equipment. Pharmacist prepares and gives medications to physician to bring into room. Second RN initiates conversation with bedside RN and starts documentation. Attending, resident, and RT don PPE and enter the room with airway equipment and ventilator. The plan for intubation is explained to the patient and a time-out is called.	T 102.1F HR 126 BP 102/64 RR 38 O2 86% on 15L NRB
12:00	Intubation.	Physician should verbalize medication dosing and avoid interventions that increase aerosolization (bagging with mask, suction, etc). RT prepares bag with a viral filter and PEEP valve, attaches to oxygen, and turns on the ventilator. If ventilator not set up prior to intubation, patient saturation will drop to 50% during intubation. Physician intubates using video laryngoscopy. ET tube immediately attached to ventilator and secured.	T 102.1F HR 130 BP 110/64 RR 36 O2 87% on 15L NRB
15:00	Hypotension after intubation (The facilitators may consider skipping this step for an early learner).	Patient becomes hypotensive and requires intervention with push dose vasoactive medications. Post-intubation sedation initiated.	T 102.1F HR 136 BP 72/40 RR vent O2 90% After pressors BP 102/64
(Case Completion)	Team coordinates for X-ray through the glass.	X-ray will arrive and request to X-ray through the door by handing plate to staff inside room. Staff will move patient and vent to avoid displacement of ET tube. Team notifies ICU of disposition.	T 102.1F HR 132 BP 110/65 RR vent O2 94%

**Table t5-jetem-5-3-s28:** 

**Critical Actions:** Team recognized the need for intubation and notifies appropriate team members, including physician, nurses, respiratory therapist, pharmacist, and an ED technician. In-room providers were limited to the physician (two if resident intubates), respiratory therapist, and nurse. Learners proceeded to intubation in a decompensating patient without bagging or other unnecessary aerosolizing procedures. Learners selected rapid sequence intubation medications such as etomidate OR ketamine for sedation and rocuronium for paralysis, and verbalized exact doses. The endotracheal tube was connected directly to the ventilator. Team anticipated and effectively treated post-intubation hypotension.	0:00

**Table t6-jetem-5-3-s28:** 

	Milestone	Did not achieve level 1	Level 1	Level 2	Level 3
1	**Emergency Stabilization (PC1)**	□ Did not achieve Level 1	□ Recognizes abnormal vital signs	□ Recognizes an unstable patient, requiring intervention Performs primary assessment Discerns data to formulate a diagnostic impression/plan	□ Manages and prioritizes critical actions in a critically ill patient Reassesses after implementing a stabilizing intervention
2	**Performance of focused history and physical (PC2)**	□ Did not achieve Level 1	□ Performs a reliable, comprehensive history and physical exam	□ Performs and communicates a focused history and physical exam based on chief complaint and urgent issues	□ Prioritizes essential components of history and physical exam given dynamic circumstances
3	**Diagnostic studies (PC3)**	□ Did not achieve Level 1	□ Determines the necessity of diagnostic studies	□ Orders appropriate diagnostic studies. Performs appropriate bedside diagnostic studies/procedures	□ Prioritizes essential testing Interprets results of diagnostic studies Reviews risks, benefits, contraindications, and alternatives to a diagnostic study or procedure
4	**Diagnosis (PC4)**	□ Did not achieve Level 1	□ Considers a list of potential diagnoses	□ Considers an appropriate list of potential diagnosis May or may not make correct diagnosis	□ Makes the appropriate diagnosis Considers other potential diagnoses, avoiding premature closure
5	**Pharmacotherapy (PC5)**	□ Did not achieve Level 1	□ Asks patient for drug allergies	□ Selects an medication for therapeutic intervention, consider potential adverse effects	□ Selects the most appropriate medication and understands mechanism of action, effect, and potential side effects Considers and recognizes drug-drug interactions
6	**Observation and reassessment (PC6)**	□ Did not achieve Level 1	□ Reevaluates patient at least one time during case	□ Reevaluates patient after most therapeutic interventions	□ Consistently evaluates the effectiveness of therapies at appropriate intervals
7	**Disposition (PC7)**	□ Did not achieve Level 1	□ Appropriately selects whether to admit or discharge the patient	□ Appropriately selects whether to admit or discharge Involves the expertise of some of the appropriate specialists	□ Educates the patient appropriately about their disposition Assigns patient to an appropriate level of care (ICU/Tele/Floor) Involves expertise of all appropriate specialists
9	**General Approach to Procedures (PC9)**	□ Did not achieve Level 1	□ Identifies pertinent anatomy and physiology for a procedure Uses appropriate Universal Precautions	□ Obtains informed consent Knows indications, contraindications, anatomic landmarks, equipment, anesthetic and procedural technique, and potential complications for common ED procedures	□ Determines a back-up strategy if initial attempts are unsuccessful Correctly interprets results of diagnostic procedure
20	**Professional Values (PROF1)**	□ Did not achieve Level 1	□ Demonstrates caring, honest behavior	□ Exhibits compassion, respect, sensitivity and responsiveness	□ Develops alternative care plans when patients’ personal beliefs and decisions preclude standard care
22	**Patient centered communication (ICS1)**	□ Did not achieve level 1	□ Establishes rapport and demonstrates empathy to patient (and family) Listens effectively	□ Elicits patient’s reason for seeking health care	□ Manages patient expectations in a manner that minimizes potential for stress, conflict, and misunderstanding. Effectively communicates with vulnerable populations, (at risk patients and families)
23	**Team management (ICS2)**	□ Did not achieve level 1	□ Recognizes other members of the patient care team during case (nurse, techs)	□ Communicates pertinent information to other healthcare colleagues	□ Communicates a clear, succinct, and appropriate handoff with specialists and other colleagues Communicates effectively with ancillary staff

## INSTRUCTOR MATERIALS

**Case Title:** An interprofessional team-based in situ simulation training for COVID PUI (Persons Under Investigation) airway preparedness

**Case Description & Diagnosis (short synopsis):** This case involves a single patient who presents to the emergency department (ED) by EMS in respiratory distress. He starts in an isolation room with a bedside nurse who performs the initial assessment. The resident and attending are alerted to a new patient roomed in their zone and review the appropriate data in the electronic medical record (Epic Playground training environment). One of the physicians may opt to enter the room upon arrival. Initial orders are placed and communication occurs with the bedside nurse about initial plan of care. The patient has signs of decompensation (respiratory distress and altered mental status) and requires intubation. The physician must activate the entire interprofessional team, including the respiratory therapist, ED pharmacist, bedside nurse, a second nurse if available, and an ED technician. The team performs a huddle outside the room to organize team members and in-room and out-of-room equipment. Personal protective equipment (PPE) is donned (verbalized, in order to conserve PPE). The inroom team sets up for intubation, administers RSI medications, and performs intubation. Intubation is complicated by hypotension (or alternatively, instructors may choose to make the complication a failed intubation). After appropriate intervention of push-dose vasoactive medications, there is an improvement of blood pressure (or placement of I-gelR/LMA in event of failed intubation). Successful endotracheal tube placement is confirmed by through-the-glass chest X-ray. Post-intubation sedation and analgesia must be verbalized, along with ventilator settings.

**Equipment or Props Needed:**

- High fidelity mannequin with IV in place
- Simulated monitor
- Gurney
- Nasal cannula
- Non-rebreather
- Surgical mask
- Room with O2 access
- Ventilator
- IV poles and pump
- Mock medications in syringes clearly labeled “for simulation use only” filled with water: etomidate, ketamine, rocuronium, phenylephrine stick, epinephrine stick, norepinephrine bag, propofol, fentanyl, versed
- Video laryngoscope with appropriate blade
- Back up Mac or Miller laryngoscope blade
- ETT (Endotracheal Tubes) 7.5 and 7.0
- Syringe
- Stylet
- Bougie
- Bag Valve Mask with PEEP valve, no mask
- Orogastric tube with syringe
- Oropharyngeal or Nasopharyngeal airway
- End-tidal CO2
- ET tube securing device
- Viral filter
- Suction Yankauer and tubing (but will avoid using if possible)
- Disposable stethoscope
- EZ-IO
- Restraints
- Mock i-STAT machine, if applicable (cardboard box prop with i-STAT illustration)
- Computers with access to Epic Playground, if applicable. A computer with PowerPoint of the electronic medical record or a Word document may be used as a substitute
- iPads, computer with video conferencing, or phones with speaker capability
- Optional: PPE if available, otherwise acceptable to verbalize

**Participants and Confederates:**

- **Participants:** Resident physician, attending physician, bedside nurse, 2nd nurse, respiratory therapist, ED Technician, ED Pharmacist
- **Confederate:** Emergency Medical Services (EMS) provider (gives report to nurse), other confederates as needed for missing professionals

**Stimulus Inventory:**

**Triage note to enter into Epic Playground:**

Pt brought in from SNF (skilled nursing facility) with fever, shortness of breath, dry cough, and fever x 1 week and confusion for x 1 day. O_2_ sats 67% this morning. Multiple +COVID contacts.
**Initial vital signs to enter:** HR 122 BP 108/66 RR 38 Sat 79% on 6L T 102.2F (39 C)
**Past Medical History:** Hypertension, Diabetes mellitus
**Medications:** Lisinopril 20mg daily, Metformin 1000mg twice daily, Enoxaparin 80mg subcutaneous every 12 hours, Acetaminophen 500mg every 6 hours as needed

*(If Epic Playground not available, please see PowerPoint slides with this information displayed as an Epic Playground screenshot).* [Table t2-jetem-5-3-s28]

**Background and brief information:** A 2-minute pre-brief will be provided prior to the start of the simulation. An introduction to the mannequin, the monitor, and the purpose of the scenario will be reviewed. We will also discuss the need to verbalize personal protective equipment (PPE) in order to conserve supplies. The individuals recruited to participate will include the resident physician, the attending physician, two nurses, a respiratory therapist, an ED technician, and a pharmacist.

The setting is an emergency department. The patient will arrive by ambulance and will be roomed immediately. The EMS provider (confederate) will provide a brief report to the bedside nurse, who will perform initial assessment. The remainder of the team will be nearby and aware that the simulation is occurring, but not yet involved in the case. The triage note and vital signs will be pre-loaded into Epic Playground. The resident and attending physician will initiate the case in the charting area and review the patient’s record in Epic Playground training environment or on word document or power point.

**Initial presentation:** The patient presents by ambulance. He was brought in from a SNF where there have been multiple other residents that have tested positive for COVID-19. He has had a cough for one week and fever and shortness of breath for one day. This morning he was found to be altered and in respiratory distress. His vital signs were checked this morning and his room air oxygen saturation was 67%; therefore 911 was called.

**How the scene unfolds:** The patient arrives by EMS (confederate) and the bedside nurse receives handoff. The physicians will have access to the triage note in Epic Playground. One physician may opt to go into the room upon patient arrival. The other physician will communicate with video conferencing software, iPad, or phone, and will place initial orders. Upon assessment, the patient will be altered with a GCS (Glasgow Coma Scale) of 13 and in significant respiratory distress. The team may escalate to nasal cannula, followed by placement of a non-rebreather mask. They may opt to trial high-flow nasal cannula, but if they do, the patient will decompensate with worsening respiratory distress and become obtunded. The physician will verbalize the need to intubate the patient. The team will be recruited and perform a huddle outside of the room. The in-room team will enter and perform intubation. The team will encounter at least one complication of intubation such as hypotension, and they will be required to address this in the scenario (option to skip this step for an early learner). The case will end with obtaining a post-intubation chest x-ray, and ICU contact for disposition, if desired.

**Critical actions:**

1. Team recognized the need for intubation and notifies appropriate team members, including physician, nurses, respiratory therapist, pharmacist, and an ED technician.
2. In-room providers were limited to the physician (two if resident intubates), respiratory therapist, and nurse.
3. Learners proceeded to intubation in a decompensating patient without bagging or other unnecessary aerosolizing procedures.
4. Learners selected rapid sequence intubation medications such as etomidate OR ketamine for sedation and rocuronium for paralysis, and verbalized exact doses.
5. The endotracheal tube was connected directly to the ventilator.
6. Team anticipated and effectively treated post-intubation hypotension.

**Case Title:** An interprofessional team-based in situ simulation training for COVID PUI (Persons Under Investigation) airway preparedness

**Chief Complaint:** Shortness of breath, fever, cough, and altered mental status arriving from a SNF.[Table t3-jetem-5-3-s28]

**General Appearance:** Lying in bed on 6L NC (Nasal Cannula) in obvious respiratory distress, speaking in 1–3 words sentences

**Primary Survey:**

- **Airway:** Intact
- **Breathing:** Rhonchi bilaterally, tachypneic
- **Circulation:** Regular tachycardic rhythm with 2+ symmetric pulses

**History:**

- **History of present illness:** 65-year-old male with history of hypertension, diabetes and recent right intramedullary nail placement for femoral fracture presents from an SNF with cough and fever and shortness of breath for one day. There have been multiple other residents that have tested positive for COVID-19 at the facility. This morning he was in respiratory distress and altered. His vital signs were checked this morning and his saturation was 67%; therefore 911 was called. He is complaining of shortness of breath, but denies chest pain, nausea, or vomiting.
- **Past medical history:** Hypertension, diabetes mellitus
- **Past surgical history:** Right intramedullary nail
- **Medications:** Lisinopril, Metformin, Enoxaparin, Acetaminophen
- **Allergies:** None
- **Social history:** Occasional alcohol use, nonsmoker, no substance use
- **Family history:** No history of asthma, COPD, or CAD
- **Code status:** Full Code

**Secondary Survey/Physical Examination:**

- **General appearance:** 65-year-old male in respiratory distress with surgical mask and NC in place
- **HEENT:**
  ○ **Head:** WNL (within normal limits), atraumatic
  ○ **Eyes:** Open, PERRL (pupils equal, round, react to light)
  ○ **Ears:** WNL
  ○ **Nose:** Nasal cannula in place covered by surgical mask
  ○ **Throat:** WNL
- **Neck:** No JVD (Jugular Vein Distention), no stridor
- **Heart:** Tachycardic, regular rhythm
- **Lungs:** Tachypneic, rhonchi bilaterally, crackles at the bases, coughing intermittently
- **Abdominal/GI:** Soft, nontender, nondistended
- **Genitourinary:** Circumcised, no scrotal swelling or pain
- **Extremities:** No swelling or deformity
- **Back:** Nontender, no deformity
- **Neuro:** GCS 13 (E3V4M6), Confused, only oriented to person. Sensation and strength intact. Unable to follow commands for ataxia testing on finger to nose.
- **Skin:** Incision to right hip clean dry and intact
- **Lymph:** WNL
- **Psych:** Appropriate mood and behavior

**Results:**

An optional i-STAT VBG and lactate may be given to the learners, if desired, but is not necessary. An optional EKG demonstrating sinus tachycardia may also be provided. Examples are provided below.

*i-STAT VBG*

Author’s own image.[Fig f1-jetem-5-3-s28]

*Electrocardiogram (EKG)*

Heilman J. Sinus tachycardia as seen on ECG. In: Wikimedia Commons. https://commons.wikimedia.org/wiki/File:Sinustachy.JPG. Published Jun 15, 2012. Accessed July 15, 2020. CC BY-SA 3.0.[Fig f2-jetem-5-3-s28]

## OPERATOR MATERIALS

[Table t4-jetem-5-3-s28]

### Diagnosis

Severe respiratory distress due to COVID-19 infection.

### Disposition

Admission to the ICU

## DEBRIEFING AND EVALUATION PEARLS

### An interprofessional team-based in situ simulation training for COVID PUI (Persons Under Investigation) airway preparedness

#### Pearls

- The main comorbidity and mortality from COVID-19 is due to acute viral pneumonitis that can develop into acute respiratory distress syndrome (ARDS).
- The decision to intubate is based on rapid progression of respiratory distress, lack of improvement with maximum noninvasive oxygenation, increased work of breathing, altered mental status, hemodynamic instability, or organ failure.
- Intubation is the procedure with highest risk for aerosolization and danger of exposure to providers in patients with COVID-19.
- Intubations should be performed in an airborne precaution isolation room if possible.
- Call for help early to assemble your team and gather equipment and resources.
- Limit number of providers in room during the high-risk aerosolizing procedure of intubation.
- Know your preferred method of communication whether through video conference or departmental phones on speaker to ensure good communication between providers inside of room and outside of room.
- Check your equipment before entering the room to limit unnecessary contamination.
- Rapid sequence intubation medications should include etomidate or ketamine and rocuronium, and appropriate weight-based doses for the patient should be drawn up prior to entering the room.
- The most experienced clinician should perform the intubation with a video laryngoscope.
- Techniques that increase aerosolization including bagging and suction should be avoided or minimized if possible.
- Have the ventilator prepared for immediate attachment to the endotracheal tube after intubation and have the bag valve mask set up and attached to oxygen in case of ventilator malfunction.
- Post-intubation hypotension should be managed with push-dose pressors and a drip should be started if needed.
- Optional: Failed intubation should be rescued with an I-gelR or alternative supraglottic airway.

#### Other debriefing points

- Closed loop communication among the team inside and outside of the room, was it used? How did this affect the management of the patient in this case?
- If the learners did not have the bag set up with a viral filter and attached to oxygen, ask the learners, “If the ventilator had malfunctioned and the patient desaturated, how would you respond?”
- Encourage contributions from all interprofessional team members; ask for their specific perspectives.

**Wrap Up:** After debriefing, participants should be thanked for their participation, and it should be emphasized that everyone’s contribution is essential in the safe execution of COVID intubations.

## SIMULATION ASSESSMENT

*An interprofessional team-based in situ simulation training for COVID PUI (Persons Under Investigation) airway preparedness*

Learner: _________________________________________

***Assessment Timeline***

This timeline is to help observers assess their learners. It allows observer to make notes on when learners performed various tasks, which can help guide debriefing discussion.[Table t5-jetem-5-3-s28]

**Critical Actions:**

□ Team recognized the need for intubation and notifies appropriate team members, including physician, nurses, respiratory therapist, pharmacist, and an ED technician.
□ In-room providers were limited to the physician (two if resident intubates), respiratory therapist, and nurse.
□ Learners proceeded to intubation in a decompensating patient without bagging or other unnecessary aerosolizing procedures.
□ Learners selected rapid sequence intubation medications such as etomidate OR ketamine for sedation and rocuronium for paralysis, and verbalized exact doses.
□ The endotracheal tube was connected directly to the ventilator. Team anticipated and effectively treated post-intubation hypotension.

**Summative and formative comments:**

**Milestones assessment:** [Table t6-jetem-5-3-s28]
