# Supplementary material for: Improving Emergency Department Airway Preparedness in the Era of COVID-19: An Interprofessional, In Situ Simulation
Source: J Educ Teach Emerg Med. 2020 Jul 15;5(3):S28–49. doi: 10.21980/J8V06M (PMC10332557; doi:10.21980/J8V06M)
Supplement: Supplementary file 1 [file jetem-5-3-s28-supp1.pptx]

## Slide 1
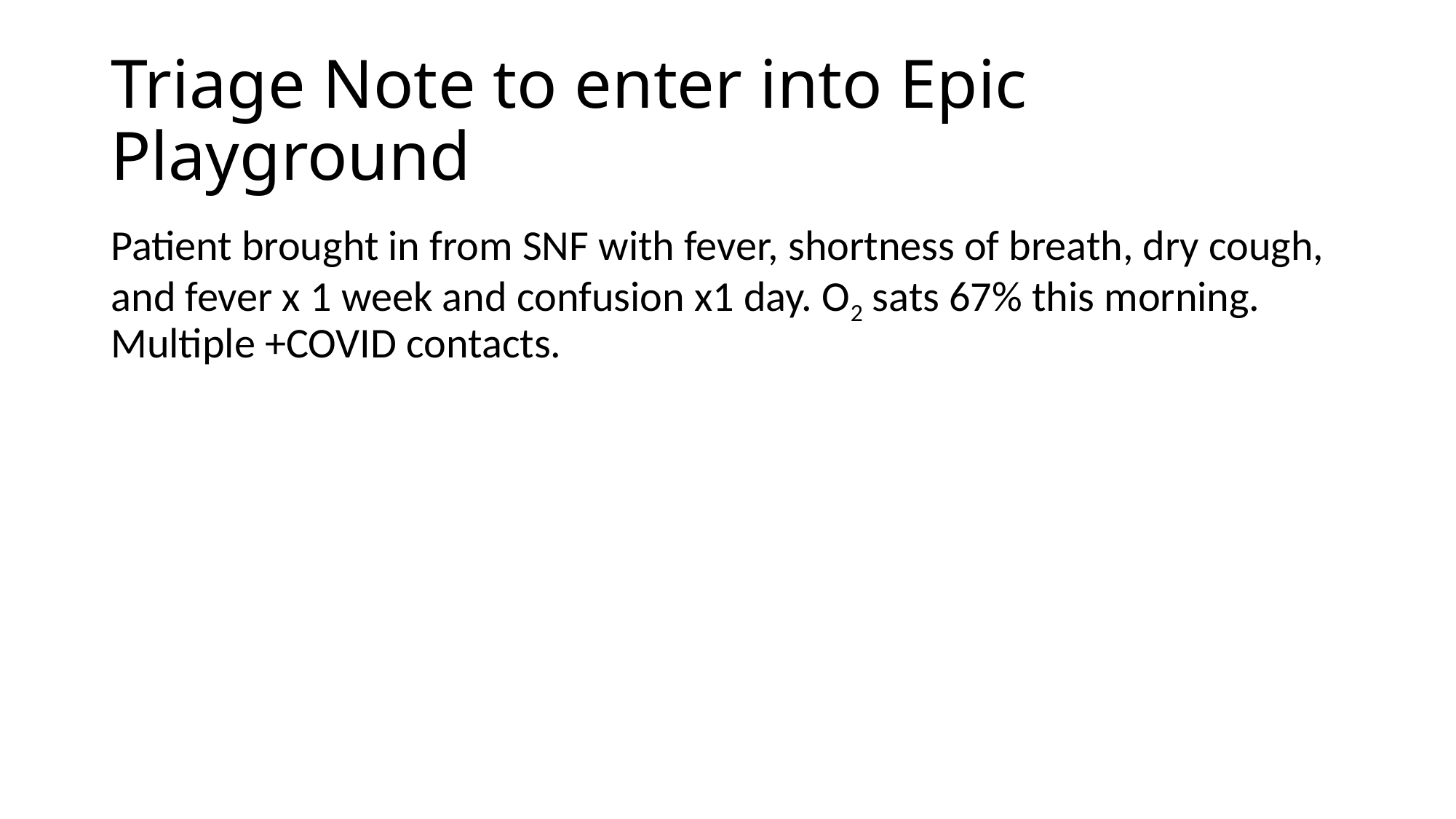

# Triage Note to enter into Epic Playground
Patient brought in from SNF with fever, shortness of breath, dry cough, and fever x 1 week and confusion x1 day. O2 sats 67% this morning. Multiple +COVID contacts.

## Slide 2
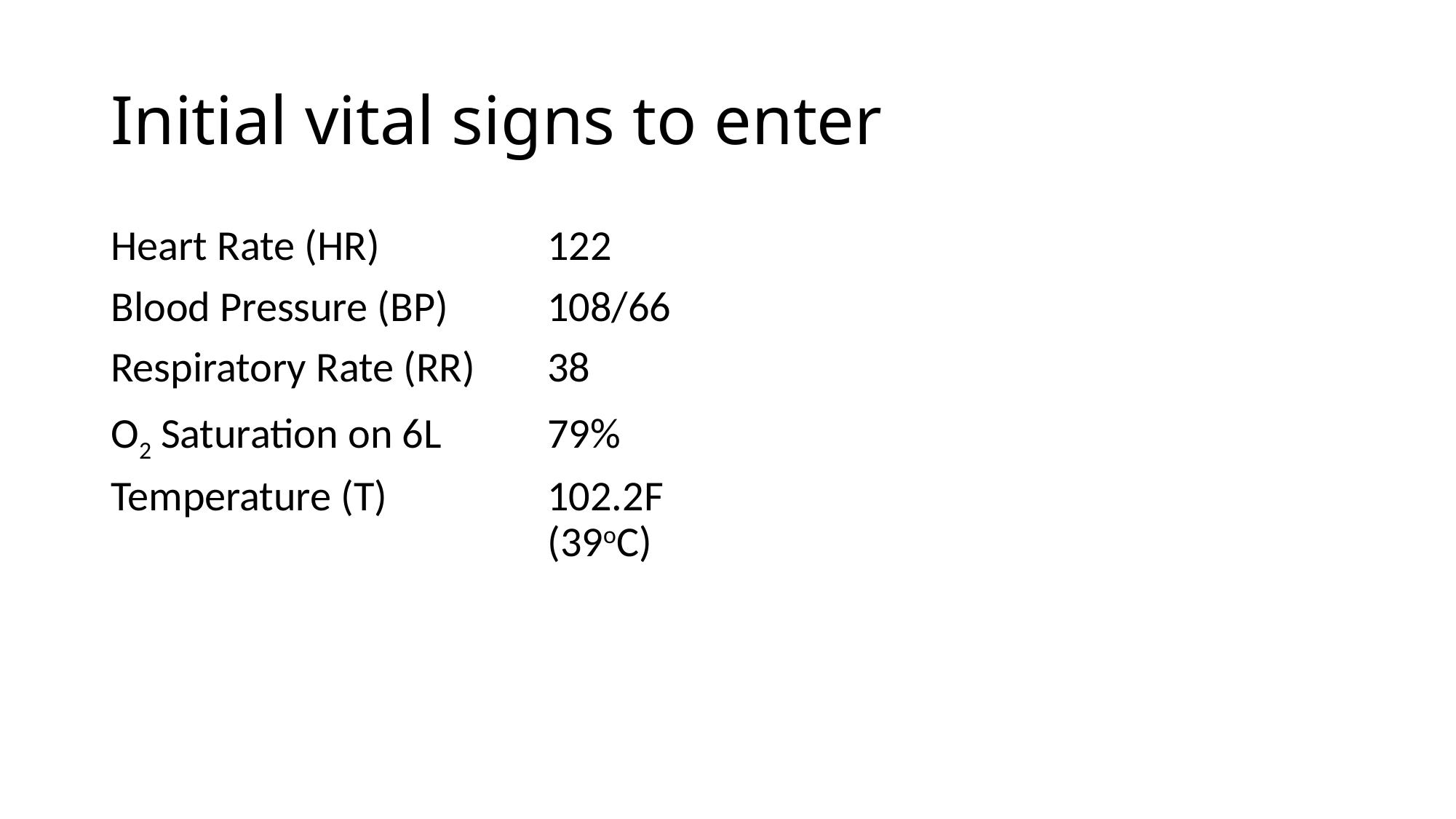

# Initial vital signs to enter
Heart Rate (HR)	 	122
Blood Pressure (BP)	108/66
Respiratory Rate (RR)	38
O2 Saturation on 6L	79%
Temperature (T)		102.2F				(39oC)

## Slide 3
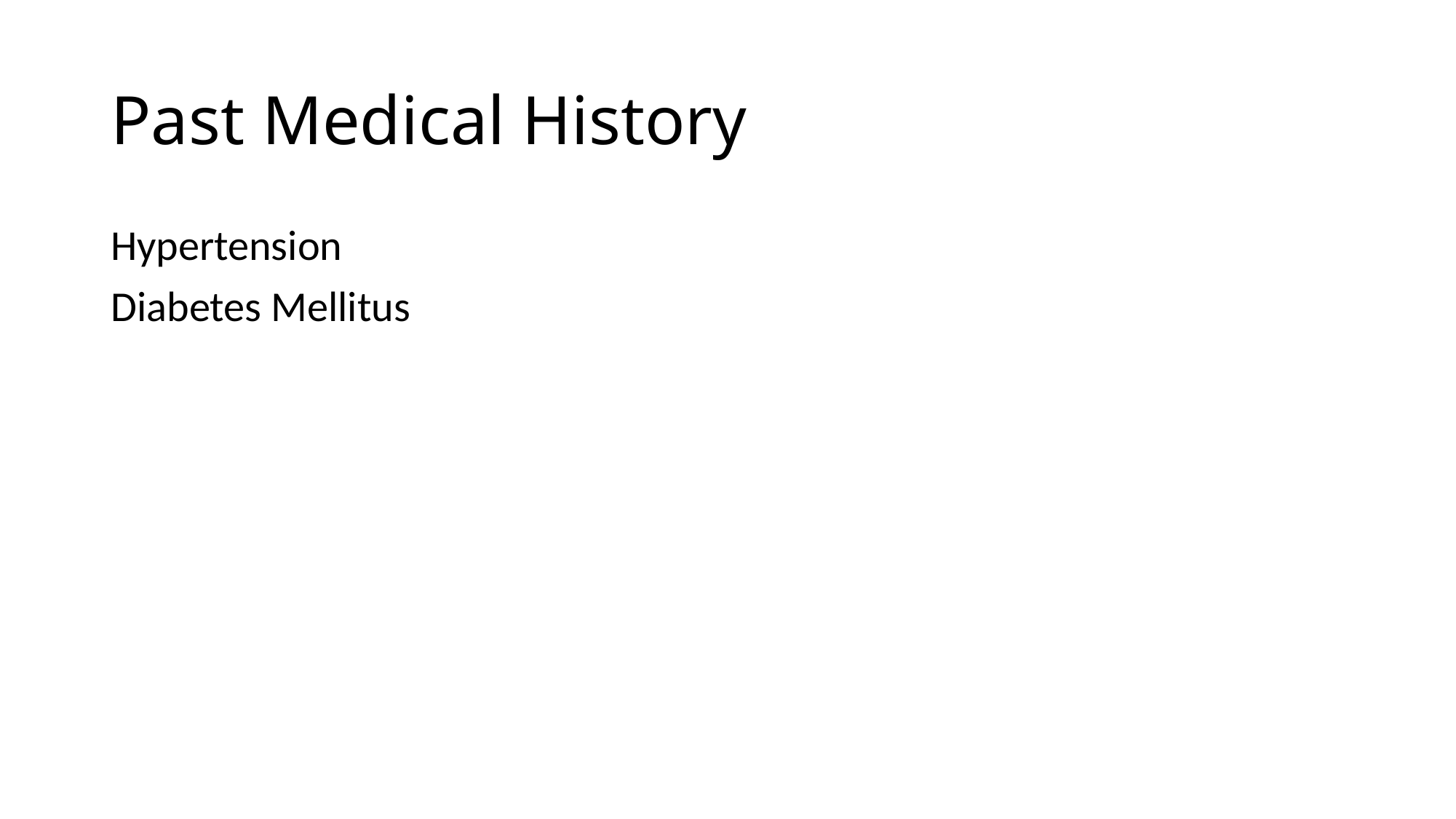

# Past Medical History
Hypertension
Diabetes Mellitus

## Slide 4
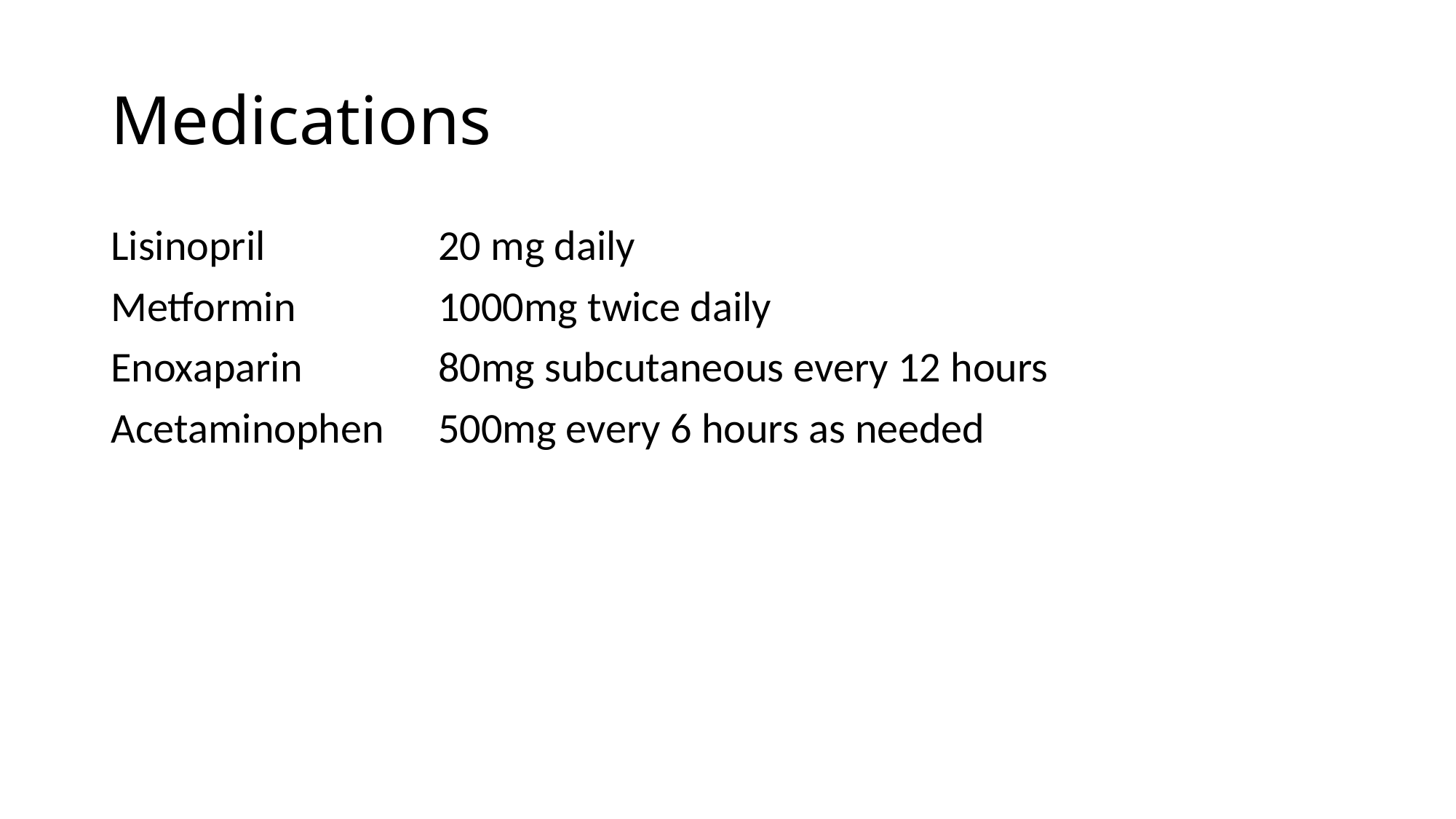

# Medications
Lisinopril		20 mg daily
Metformin		1000mg twice daily
Enoxaparin 		80mg subcutaneous every 12 hours
Acetaminophen 	500mg every 6 hours as needed

## Slide 5
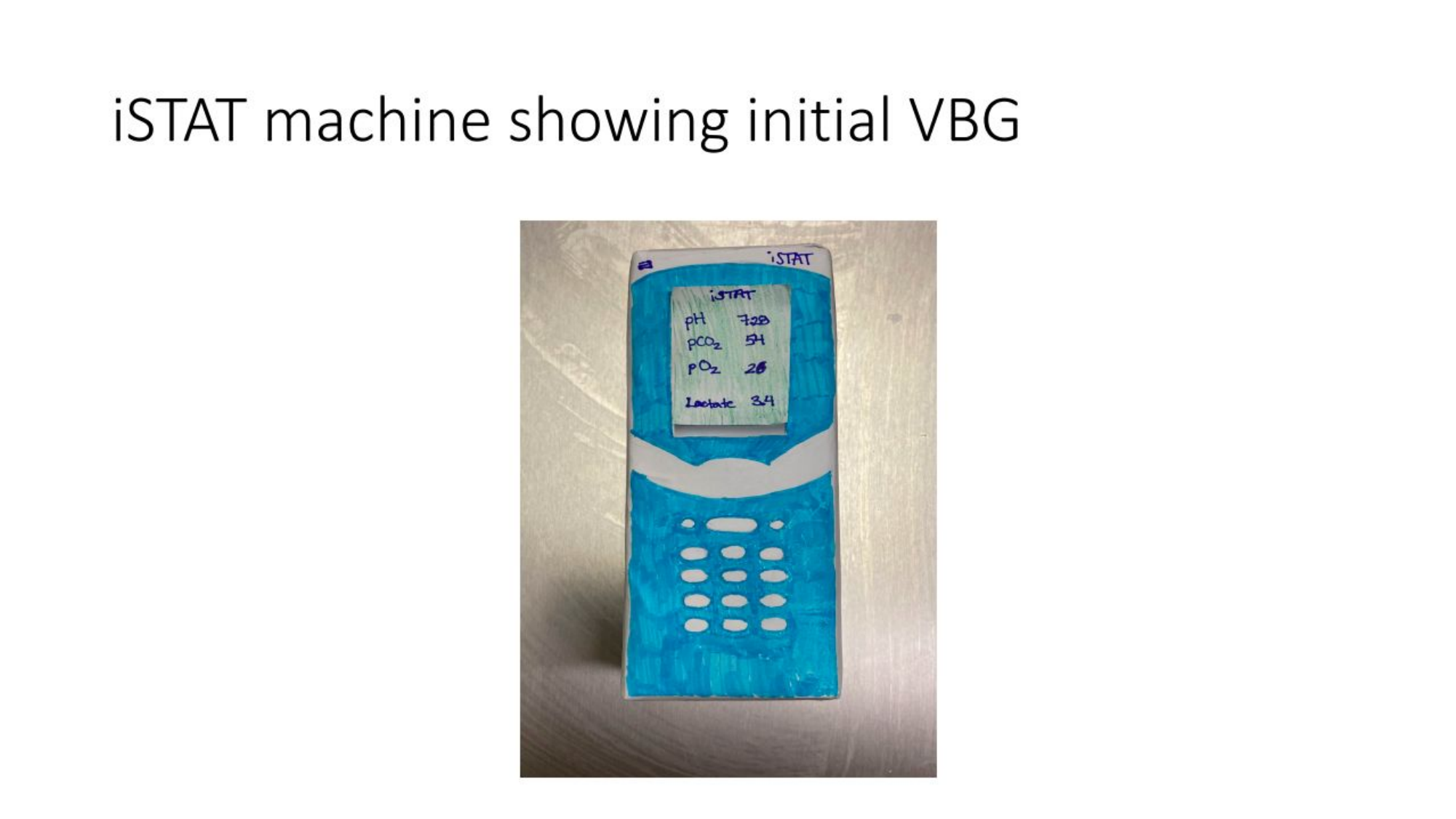

# iSTAT machine showing initial VBG

## Slide 6
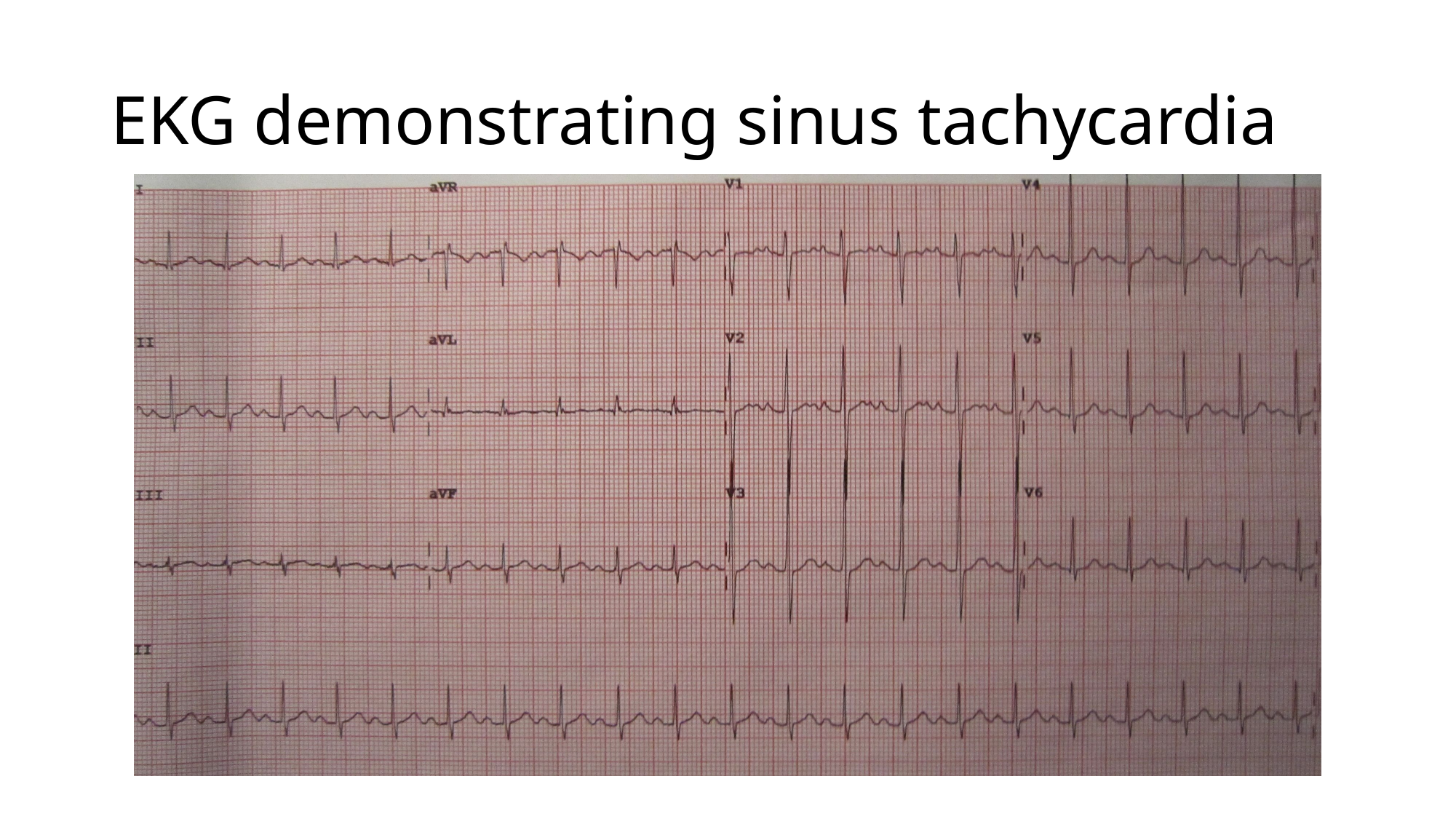

# EKG demonstrating sinus tachycardia
