# Supplementary material for: Improving Emergency Department Airway Preparedness in the Era of COVID-19: An Interprofessional, In Situ Simulation
Source: J Educ Teach Emerg Med. 2020 Jul 15;5(3):S28–49. doi: 10.21980/J8V06M (PMC10332557; doi:10.21980/J8V06M)

## Slide 1
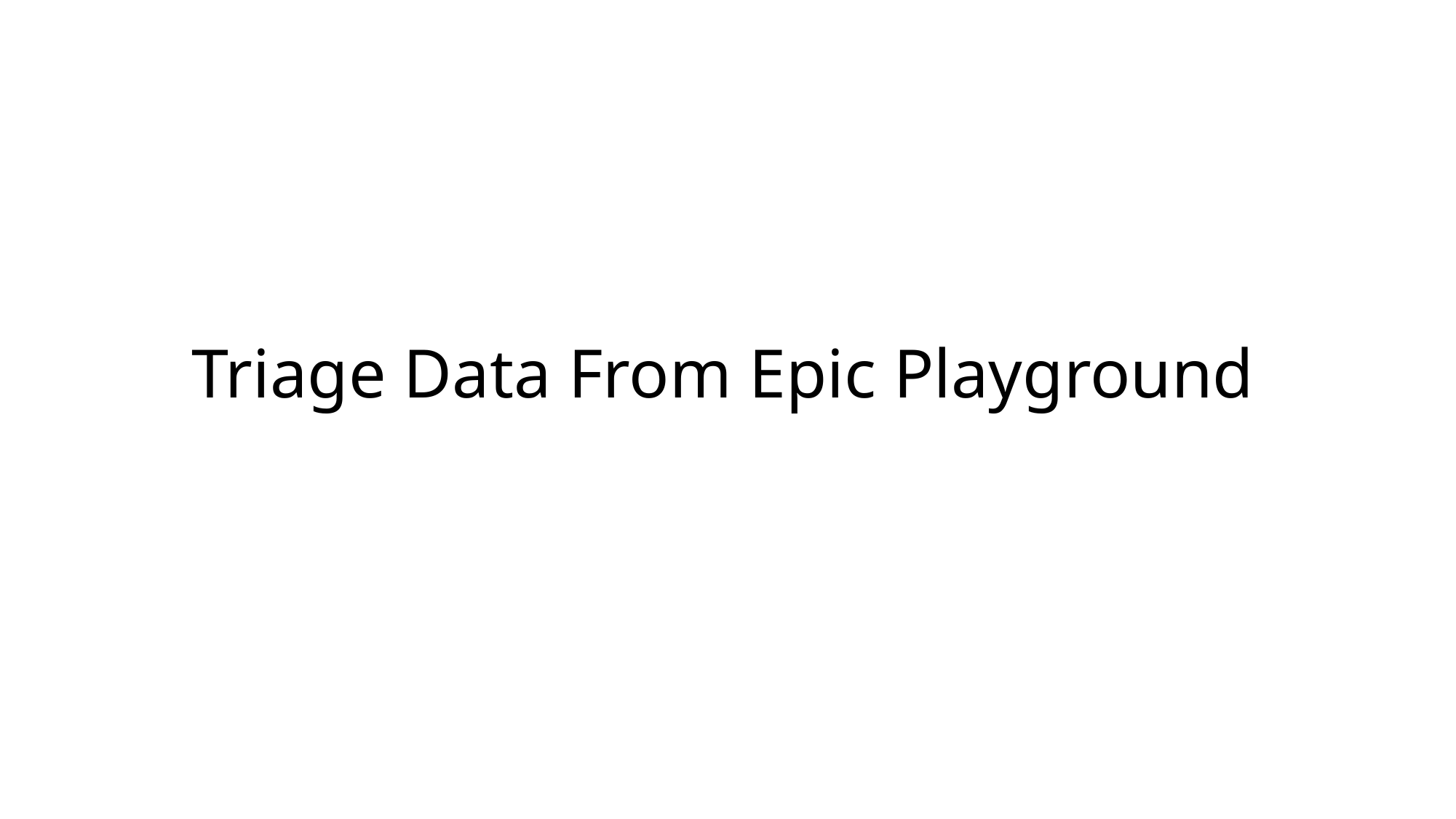

# Triage Data From Epic Playground

## Slide 2
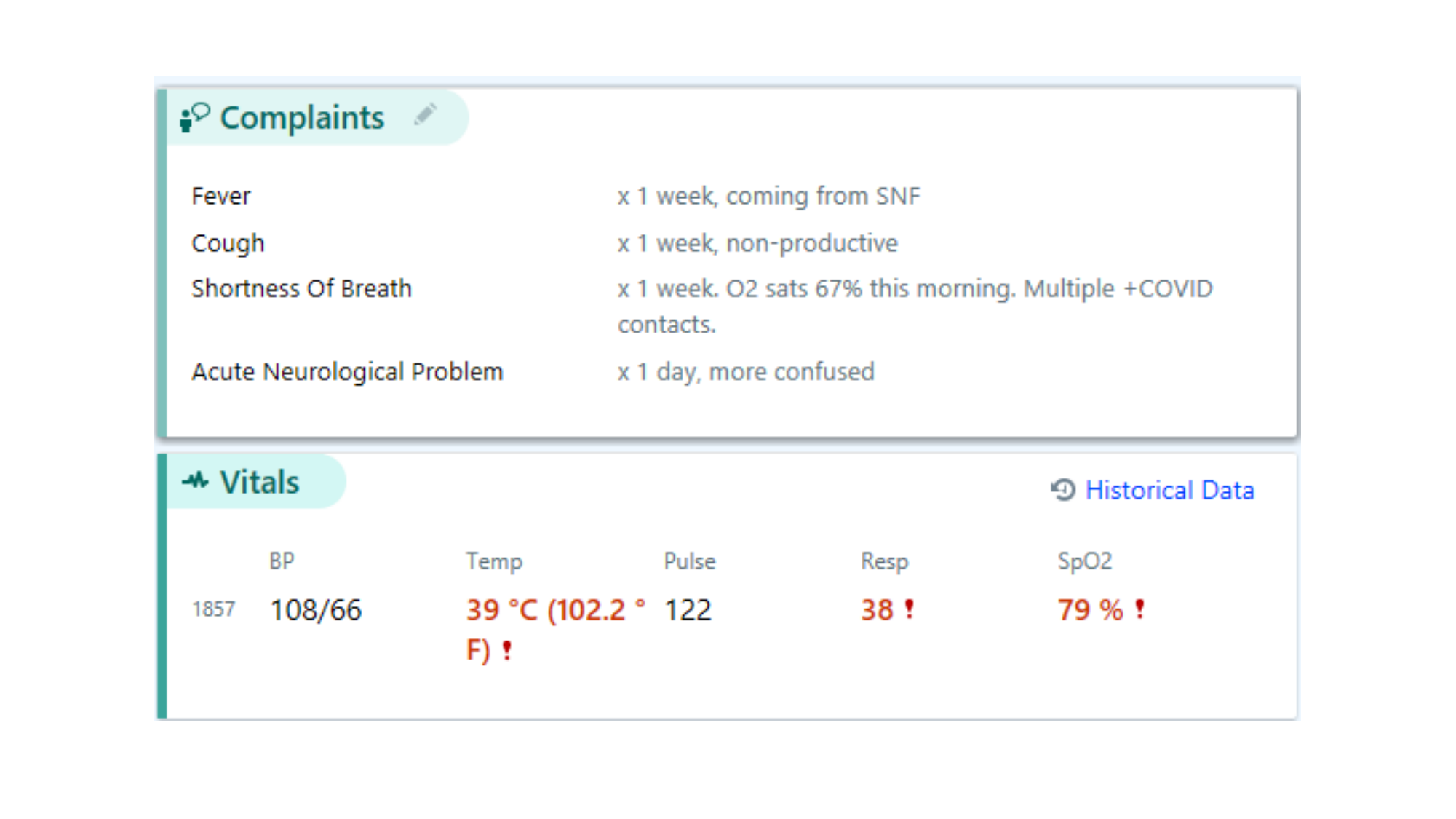

#

## Slide 3
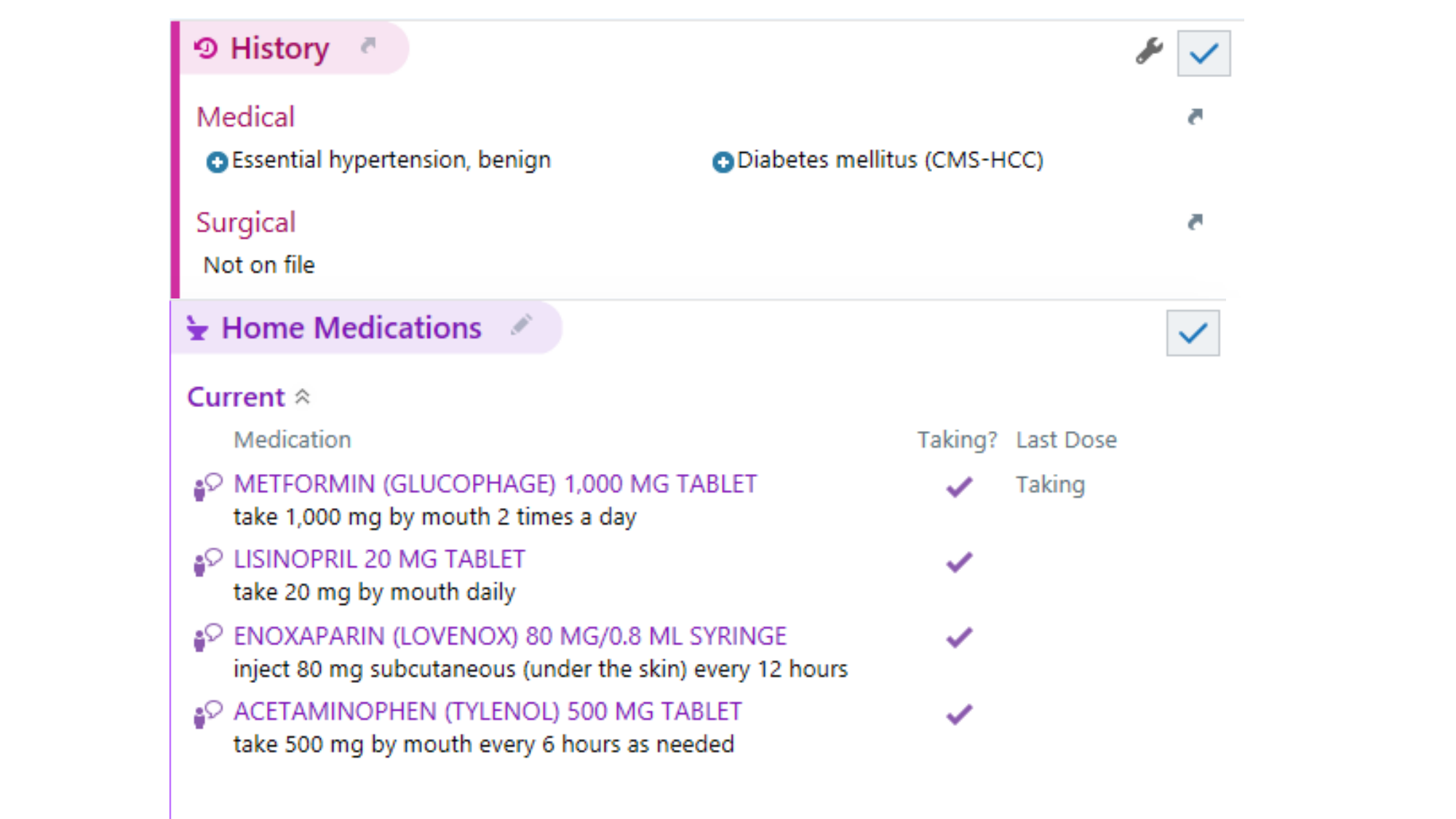

## Slide 4
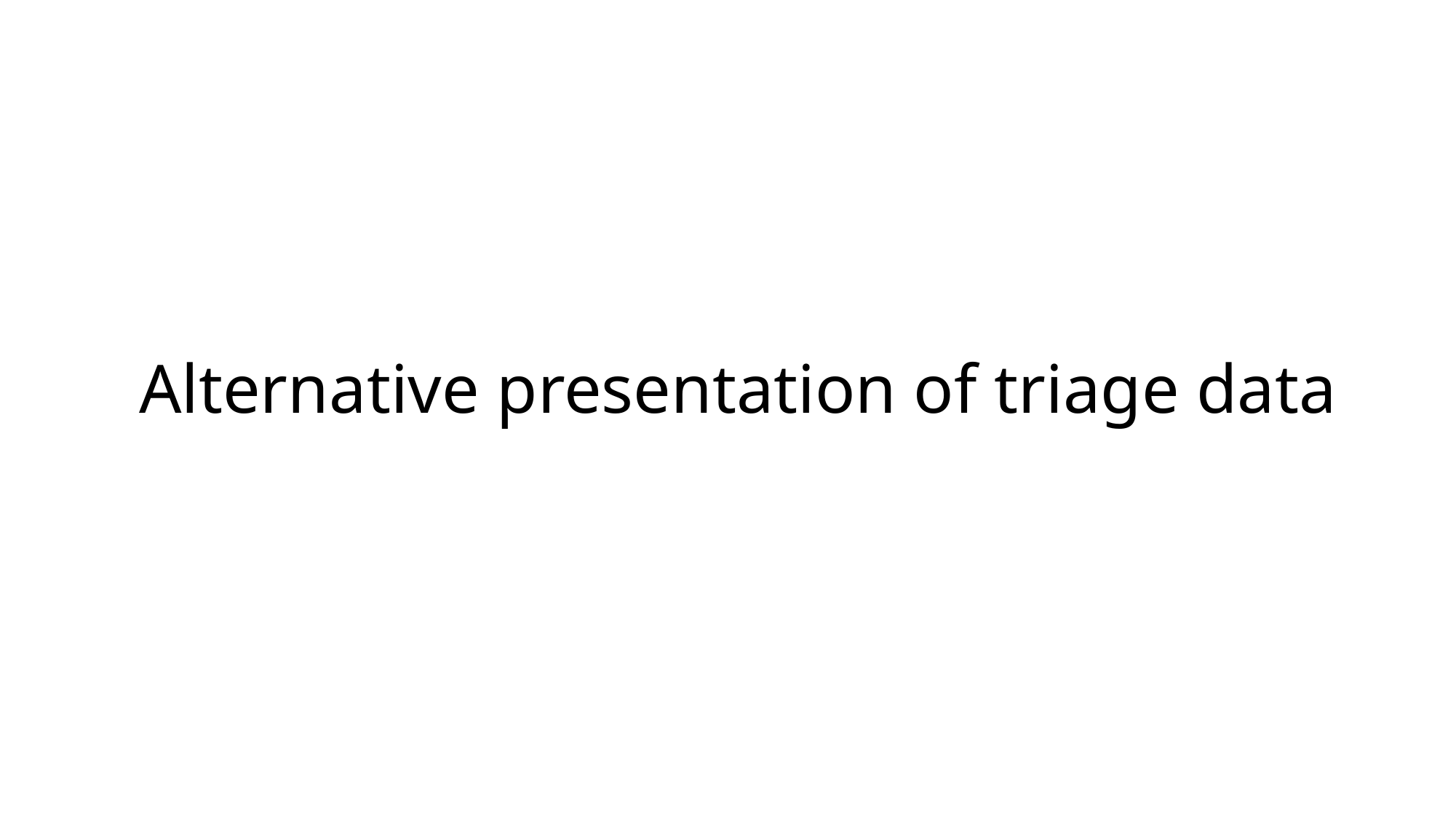

# Alternative presentation of triage data

## Slide 5
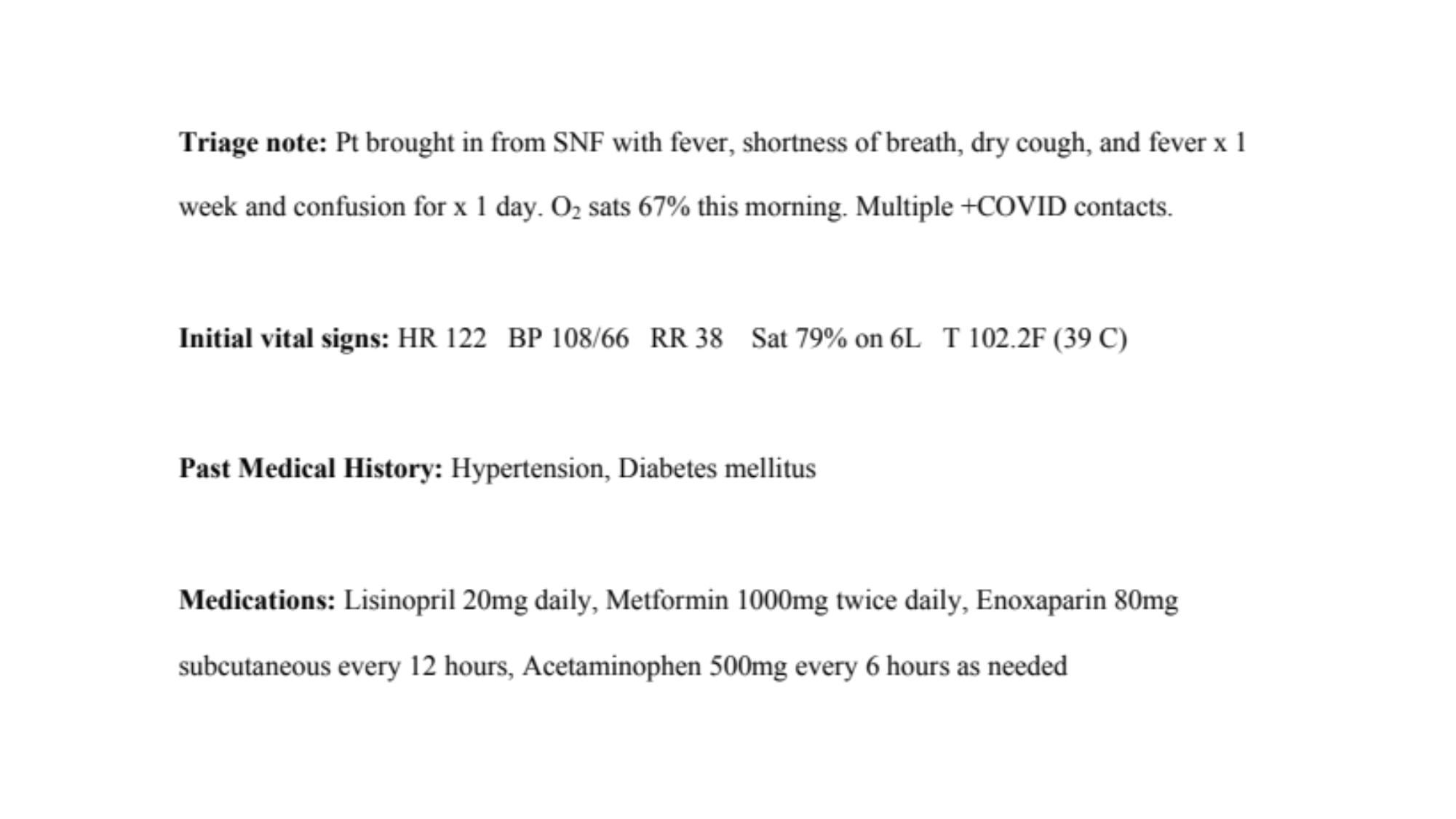

Supplement: Supplementary file 2 [file jetem-5-3-s28-supp2.pptx]
